# Engineered Disease Resistance in Cotton Using RNA-Interference to Knock down *Cotton leaf curl Kokhran virus-*Burewala and Cotton leaf curl Multan betasatellite Expression

**DOI:** 10.3390/v9090257

**Published:** 2017-09-14

**Authors:** Aftab Ahmad, Muhammad Zia-Ur-Rehman, Usman Hameed, Abdul Qayyum Rao, Ammara Ahad, Aneela Yasmeen, Faheem Akram, Kamran Shahzad Bajwa, Jodi Scheffler, Idrees Ahmad Nasir, Ahmad Ali Shahid, Muhammad Javed Iqbal, Tayyab Husnain, Muhammad Saleem Haider, Judith K. Brown

**Affiliations:** 1 Center of Excellence in Molecular Biology, University of the Punjab, 87 W Canal Bank Road, Thokar Niaz Baig, Lahore 53700, Pakistan; ammara_ahad@yahoo.com (Am.A.); anilla_malik5@hotmail.com (A.Y.); faheem_bot@yahoo.com (F.A.); dr.kamran.molebiol@gmail.com (K.S.B.); dr.idrees@gmail.com (I.A.N.); ahmadali.shahid@googlemail.com (A.A.S.); tayyabhusnain@yahoo.com (T.H.); 2 Institute of Agricultural Sciences (IAGS), University of the Punjab, Lahore 54590, Pakistan; zmansbs@gmail.com (M.Z.-U.-R.); usman_sbs@yahoo.com (U.H.); javed786_pk@yahoo.com (M.J.I.); haider65us@yahoo.com (M.S.H.); 3 Jamie Whitten Delta States Research Center, United States Department of Agriculture (USDA), Stoneville, MS 38776, USA; Jodi.Scheffler@ars.usda.gov; 4 School of Plant Sciences, University of Arizona, Tucson, AZ 85721, USA; jbrown@cals.arizona.edu

**Keywords:** *Begomovirus*, cotton leaf curl disease, Rep protein, siRNA, transgenic resistance

## Abstract

Cotton leaf curl virus disease (CLCuD) is caused by a suite of whitefly-transmitted begomovirus species and strains, resulting in extensive losses annually in India and Pakistan. RNA-interference (RNAi) is a proven technology used for knockdown of gene expression in higher organisms and viruses. In this study, a small interfering RNA (siRNA) construct was designed to target the *AC1* gene of *Cotton leaf curl Kokhran virus*-Burewala (CLCuKoV-Bu) and the *βC1* gene and satellite conserved region of the Cotton leaf curl Multan betasatellite (CLCuMB). The *AC1* gene and CLCuMB coding and non-coding regions function in replication initiation and suppression of the plant host defense pathway, respectively. The construct, *Vβ*, was transformed into cotton plants using the *Agrobacterium*-mediated embryo shoot apex cut method. Results from fluorescence in situ hybridization and karyotyping assays indicated that six of the 11 T_1_ plants harbored a single copy of the Vβ transgene. Transgenic cotton plants and non-transgenic (susceptible) test plants included as the positive control were challenge-inoculated using the viruliferous whitefly vector to transmit the CLCuKoV-Bu/CLCuMB complex. Among the test plants, plant Vβ-6 was asymptomatic, had the lowest amount of detectable virus, and harbored a single copy of the transgene on chromosome six. Absence of characteristic leaf curl symptom development in transgenic Vβ-6 cotton plants, and significantly reduced begomoviral-betasatellite accumulation based on real-time polymerase chain reaction, indicated the successful knockdown of CLCuKoV-Bu and CLCuMB expression, resulting in leaf curl resistant plants.

## 1. Introduction

Cotton leaf curl disease (CLCuD) is caused by a number of different begomoviral species and strains that infect a wide range of plant genera, including cotton, *Gossypium* spp., many of which are members of the Malvaceae. In Pakistan, crop losses due to leaf curl disease have ranged between 5% and 30% or more since 1990, having an estimated worth of US$5 billion. During the 2008–2009 outbreak, losses amounted to ~1.12 million bales, affecting over 1.48 million hectares [1,2,3]. Even though Pakistan is the third- and fourth-largest exporter and producer of cotton respectively, worldwide, it ranks thirteenth in yield per acre, requiring the importation of 1.5–2.00 million bales of cotton each year to support the capacity of textile mills [4].

Despite the widespread use of transgenic solutions to pest control in cotton—for example, Bt-toxin expression to reduce feeding damage by lepidopteran pests—and the robust evidence showing that pathogen-derived resistance based on gene silencing is effective for combatting diseases caused by plant viruses [5,6,7], the commercialization of transgenic technology for virus disease control has been limited [3]. In particular, RNA-interference (RNAi) [8], induced by double-stranded (ds) RNA, has been shown highly effective for silencing viral gene expression in plants to achieve disease resistance, including for geminiviruses [9,10,11,12,13]. Stable knockdown of viral gene expression has been demonstrated using small interfering RNA (siRNA) technology by targeting viral coding and non-coding regions involved in viral replication and movement, which are crucial for establishing system infection of the host plant [8,11]. The knockdown effect of RNAi spreads systemically throughout the plant, insects, and in certain other organisms harboring RNAi machinery, the dsRNA is exported to neighboring cells [7]. Thus, RNAi is among the most promising technologies for developing virus-resistant crops through transgenic expression of double-stranded RNA [4]. This application of RNAi could help alleviate the overwhelming reliance on chemical pesticides for controlling insect vectors [14], such as the whitefly, *Bemisiatabaci* (Genn.), which transmits begomoviruses [15,16], while also reducing rates of virus transmission.

Begomoviruses (family, *Geminiviridae*; genus, *Begomovirus*) are small, single-stranded DNA viruses that are pathogens of cultivated and non-cultivated wild plant species, nearly worldwide. They have either a bipartite (DNA-A and DNA-B) or monopartite (DNA-A) genome organization. Many monopartite begomoviruses require an associated betasatellite to facilitate systemic infection of the plant host, in part by suppressing plant host defenses [17,18,19]. The begomoviral*AC1* gene, or replication (Rep)-associated protein, encoded on all DNA-A components, is a multifunctional protein that localizes to the plant nucleus. The *AC1* is essential for regulating begomoviral transcription and replication of viral DNA [20,21] following the introduction of a nick at the origin of replication (*ori*). The Rep-associated protein also interacts with sequences in the satellite-conserved region (SCR) of the betasatellite, or DNA β-satellite [20] to facilitate its replication. The DNA β-satellite SCR shares an identical nonanucleotide sequence with its respective “helper virus”, suggesting this non-coding region is essential for β-satellite replication initiation. The SCR and its position in the DNA β-satellite molecule appears to be analogous the common region position of bipartite begomoviruses [18].

Begomovirus-associated β-satellites modulate begomovirus virulence, and therefore symptom development and severity [22]. In plants inoculated with the helper virus DNA-A, in the absence of the DNAβ satellite, the virus accumulates to much lower levels compared to wild type virus accumulation, and symptoms are attenuated or entirely absent [19,22]. The only coding region known to occur on DNA β-satellites, is referred to as *Beta C1* (*βC1*), which codes for a protein that functions as a suppressor of post-transcriptional gene silencing, and so is considered to be a key determinant of pathogenicity. Silencing of *βC1* expression therefore is expected to interfere with begomoviral systemic infection of the host plant [19,22,23].

Here, a dsRNA (hairpin) was constructed, consisting of a cloned fragment of the *AC1* gene *Cotton leaf curl Kokhran virus*-Burewala (CLCuKoV-Bu; HF567942), and a fragment of the *βC1* coding region overlapping with the non-coding SCR of Cotton leaf curl Multan betasatellite (CLCuMB; HF567946). The sequence integrity of the fragments following sub-cloning was verified by confirmatory DNA sequencing, and the construct was transformed into *Gossypiumhirsutum* “VH-289”, a cotton variety that is adapted for cultivation in Pakistan.

## 2. Materials and Methods

### 2.1. Plasmid Construction for siRNA/Intron Spliced Hairpin RNA Generation

**Figure 1:**
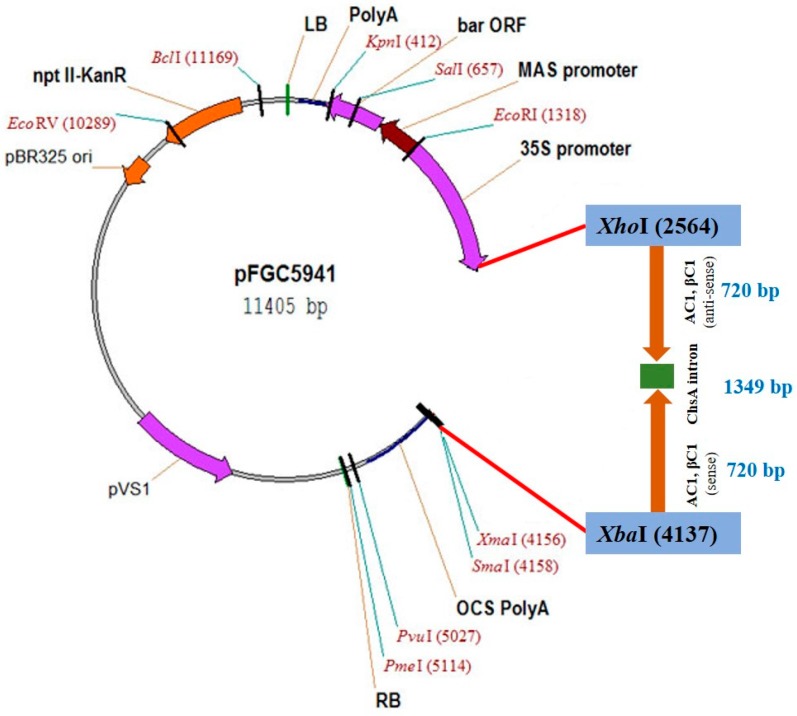
Map of the Vβ construct.

The RNAi construct, herein designated Vβ, was designed to target coding and non-coding regions of the CLCuKoV-Bu *AC1* gene and CLCuMB DNA *βC1*. The targets were selected on the basis of their requirement for viral replication and satellite-mediated suppression of host-defenses. The hairpin construct was made by cloning a 720 bp fragment containing the target sequences in the plasmid vector pFGC5941 [5], in the sense and anti-sense orientations separated by a 1349 bp fragment of the chalcone synthase A intron (ChsA), previously cloned from *Petunia × hybrida; hort. ex E.Vilm.* [24]. The plasmid vector contains the constitutive 35S CaMV-promoter to drive expression, and the octopine synthase (OCS) terminator (Figure 1).

### 2.2. Cotton Plant Transformation

Seeds of the cotton variety “VH-289” were obtained from the Central Cotton Research Institute (CCRI, Multan, Pakistan). The delinted seeds were held in high moisture conditions for germination, and plant transformation was carried out, as previously described [25]. In addition, some non-transformed embryos were plated on MS medium to create a transformation-minus control. The putative, transgenic cotton plants were selected using Basta herbicide also known asglufosinate-ammonium a broad-spectrum systemic herbicide (Bayer Crop Science, Thane, Maharashtra, India)at 200 mg/L, for two-months, and the non-transgenic plants were cultured on rooting and shooting medium, with and without Bastaselection. The plantlets surviving herbicide selection, and non-transgenic control plantlets which survived on medium without Basta selection, were transferred to pots containing a potting mix, and acclimatized to greenhouse conditions. The seeds were collected from positively transformed plants, confirmed by PCR amplification and sequencing of amplicons, planted, and grown under controlled conditions to produce the T_1_ generation seed.

### 2.3. Molecular Analysis of Transgenic Cotton Plants

Total DNA was purified from the emerging leaves of transgenic and non-transgenic plants using the CTAB (cetyltrimethylammonium bromide) method [26]. Positively transformed plants were identified by PCR amplification of a fragment of CLCuKoV-Bur *AC1* using the specific primers, F-5′-TGCCAAAAACTATTTCCTCACAT-3′ and R-5′-AACGTCTCCATCTTTGGCG-3′, to obtain an expected size product of 301 bp, as described previously [27].

### 2.4. Challenge-Inoculation of Transgenic Plants

Resistance to CLCuD in T_0_ and T_1_ (generation 0 and 1) transgenic cotton plants was evaluated on the basis of symptom development, and disease severity rating score, as described previously [28]. Adult whiteflies, *B. tabaci* (Asia II major clade mitotype), were allowed a four-day acquisition access period (AAP) on cotton plants infected with CLCuKoV-Bur and CLCuMB and transferred to 4–6 leaf stage transgenic and non-transgenic cotton seedlings, at the 3–4 leaf stage, for a four-day inoculation access period (IAP). Whiteflies were killed by insecticide treatment. Plants were maintained an insect-free greenhouse, and observed periodically for symptom development, and observations were recorded, three weeks post-inoculation.

### 2.5. Real-time Polymerase Chain Reaction Analysis

Virus accumulation in virus-inoculated, transgenic cotton plants harboring the dsRNA hairpin construct designed to silence CLCuKoV-Bur/CLCuMB gene expression, was quantified using the Thermo Scientific Maxima SYBR Green qPCR kit (cat# K0241) (Thermo Fisher Scientific, Waltham, MA, USA). Total DNA was isolated from cotton plants, diluted 10-fold, and used as a template for quantitative, real-time PCR amplification. A cloned, full-length CLCuMB molecule was used as the baseline reference sequence for quantification. Total DNA isolated from a cotton plant infected with CLCuKoV-Bur/CLCuMB, or from a virus-free plant and included as positive and negative experimental controls, respectively. Cycling conditions were: 5 min at 95 °C for 40 cycles, 30 s at 95 °C, and 30 s at 55 °C. The melting curve was produced by denaturing the amplicon in a temperature gradient of 60 to 95 °C to confirm specificity of the CLCuMB primers, βF-5-AGTGCGCTGAAAAAGGTGAT-3′ and βR-5-ATTAAAACGTGAAAAAGGTGAT-3′. The fold-change was calculated by comparing the normalized transcript levels reflecting viral-betasatellite gene expression in transgenic plants, results obtained for DNA from control plants.

### 2.6. Fluorescence In Situ Hybridization

Using the Fluorescein ULS^®^ Labeling Kit Cat. No. K0641 (Fermentas, Thermo Science Fisher), the probe for transgene detection was labeled according to the manufacturer’s instructions. In situ hybridization was performed, as previously described [29] for metaphase chromosomal spreads [12], with the counterstaining of hybridized slides, copy number determination, and transgene localization on cotton chromosomes, as previously described [9,29,30].

### 2.7. In Silico Analysis of dsRNA Hairpin Structure

The complete sequences of viral *AC1* and *βC1* genes, and the non-coding SCR betasatellite region of CLCuKoV-Bur and CLCuMB were targeted using RNAi. The sense and anti-sense sequences for each of the target regions used to build the construct were obtained by PCR amplification and cloning into the plasmid vector pFGC5941. The “spacer”, or the sequence used to achieve loop formation was a previously inserted (engineered) fragment of the *ChsA* gene.

The DNA sequences were converted to RNA using the Sequence Conversion tool [31] available at In-silico.net. The RNA sequences were analyzed using the on-line RNA secondary structure prediction tool, RNA structure [32] available at Predict a Secondary Structure Web Server [33].

## 3. Results

### 3.1. Genetic Transformation of Cotton

Transformation of the cotton variety “VH-289” with the Vβ construct was done using the embryo shoot apex cut method, as previously described [14]. Two days post co-cultivation the cotton plantlets were transferred to selection media, containing 200 mg/L Basta (Bayer Crop Science) for positive selection screening, with the addition of phytohormoneindole-3-butyric acid (IBA) (0.1 mg/L) to facilitate root establishment.The efficiency of transformation was 2.8% (Table 1). After the plants were acclimatized to greenhouse conditions, leaf samples were collected from cotton plants, and used for molecular analysis and karyotyping.

### 3.2. Confirmation of Transgene Presence in T_0_ and T_1_ Plants

**Figure 2:**
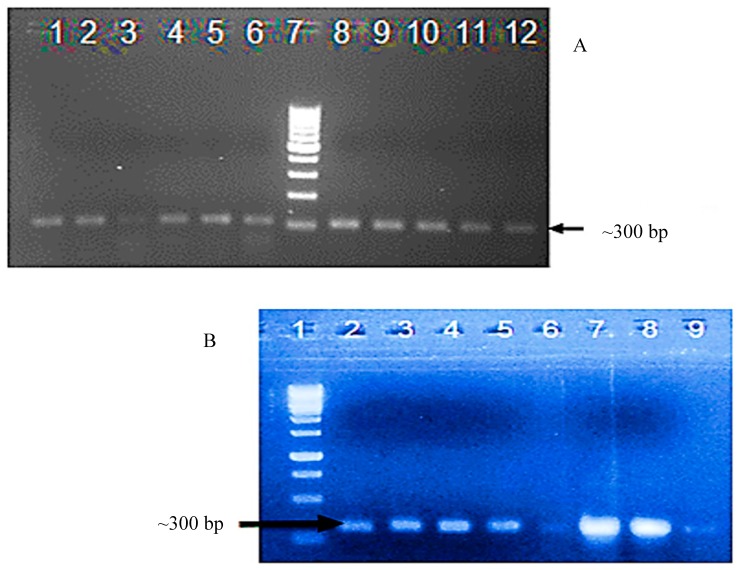
(**A**) Detection of transgene integration in the T_0_ generation of transgenic cotton plants by polymerase chain reaction amplification using *AC1* gene specific primers, which yielded the expected size ~300 bpamplicon. Lanes 1–5: PCR amplification from transgenic cotton plants; Lane 6 and 8: positive controls (full-length CLCuKoV-Bur DNA-A clone); Lane 7: 1kb ladder; and Lanes 9–12: PCR products amplified from transgenic cotton plants. (**B**) Detection of transgene integration in DNA isolated from T_1_ generation transgenic cotton plants by polymerase chain reaction (PCR) using CLCuKoV-Bur*AC1*-specific primers, which yielded the expected size ~300 bp amplicon, and confirmatory DNA sequencing. Lane 1: 1kb ladder; Lane 2: positive control, full-length CLCuKoV-Bur DNA-A clone); Lanes 3–9: PCR products amplified from transgenic cotton plants.

Confirmatory PCR amplification using viral *AC1*-specific primers was carried out to confirm transformation of the 11 resultant cotton plants. The expected size ~300 bp fragment, corresponding to a region of *AC1*, was amplified from the nine T_0_ cotton plants that survived acclimatization. Seven of the T_1_ transgenic cotton plants were positive, based on PCR amplification, which yielded the expected size band of ~300 bp, cloning, and confirmatory DNA sequencing (Figure 2). 

### 3.3. Challenge Inoculation of Transgenic Plants with CLCuKoV-Bu/CLCuMB by Whitefly Inoculation

The challenge-inoculation study was carried out using adult, viruliferous whiteflies (10 per plant) to inoculate the greenhouse-maintained transgenic and non-transgenic (negative control) cotton plants with CLCuKoV-Bu/CLCuMB. Characteristic CLCuKoV-Bu/CLCuMB symptoms developed in all of the non-transgenic plants (Table 2), three weeks post-inoculation. However, leaf curl symptoms were not observed in the transgenic T_0_ and T_1_ cotton plants, with one exception, line T1Vβ4 (T_1_), which exhibited mild leaf curl symptoms, receiving a symptom severity score of 1. A symptom severity index was implemented to assign a disease severity score to each test plant. The severity index score virus-infected, non-transgenic, positive control plants was 83.3%, whereas, the T_0_ and T_1_ transgenic plant scores were 0% and 4.16%, respectively.

### 3.4. Betasatellite Accumulation in Transgenic and Non-Transgenic Cotton Plants, Post-Virus Inoculation Using Viruliferous Whiteflies

**Figure 3:**
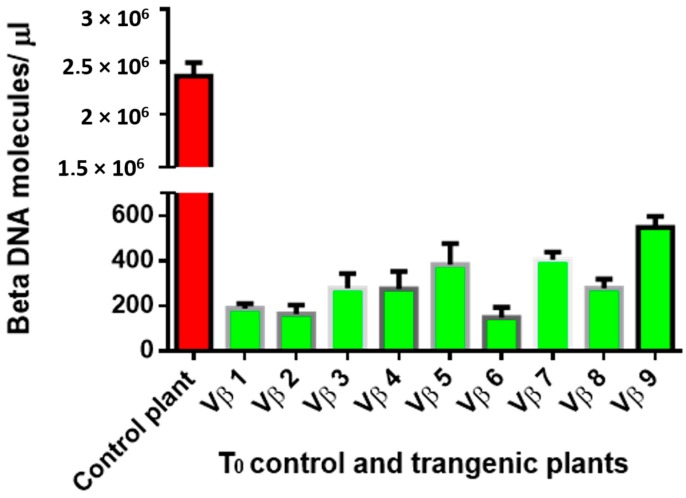
Quantitative polymerase chain reaction amplification detection of CLCuMB molecules/µLin total DNA purified from the nine T_0_ transgenic cotton plants (Vβ1–Vβ9), compared to a non-transgenic, positive control cotton plant.

**Figure 4:**
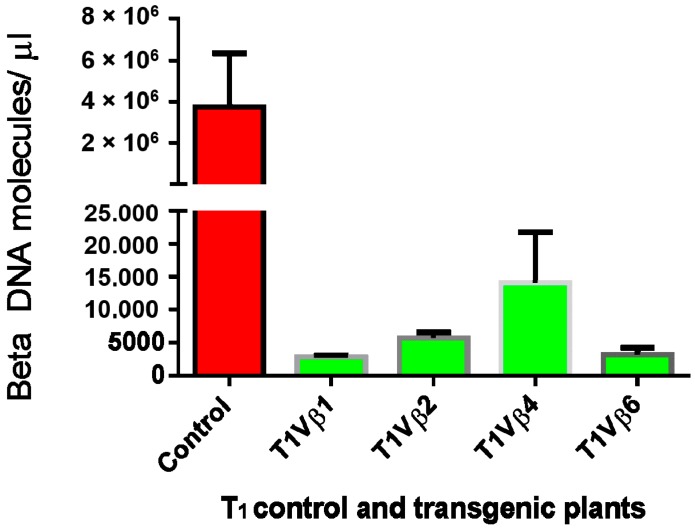
Quantitative polymerase chain reaction amplification detection of CLCuMB molecules/µL in total DNA purified from the T_1_ transgenic cotton plants, T1Vβ1, T1Vβ2, T1Vβ4, T1Vβ6, compared to a non-transgenic, positive control cotton plant.

**Figure 5:**
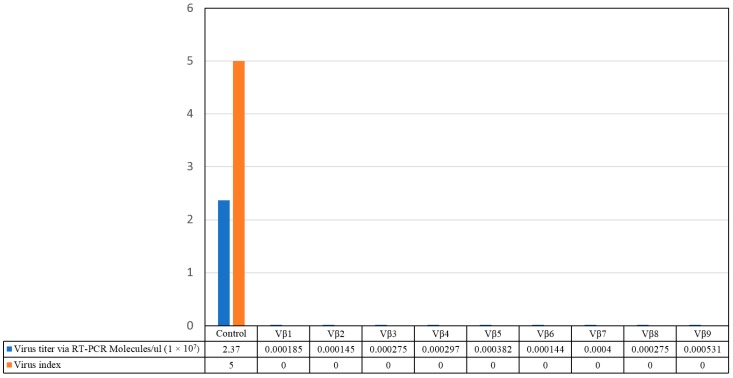
Comparison of virus disease severity score with virus accumulation in the T_0_ transgenic cotton plants, lines Vβ1–Vβ9, and a non-transgenic, positive control cotton plant.

**Figure 6:**
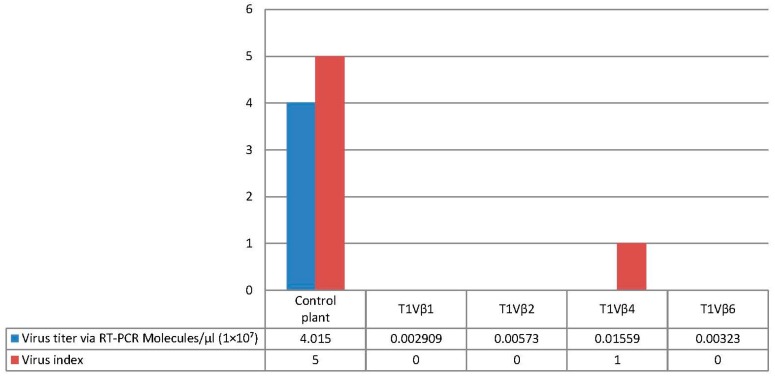
Comparison of disease severity score and virus accumulation in the T_1_ transgenic cotton plants, lines T1Vβ1, T1Vβ2, T1Vβ4, T1Vβ6, and a non-transgenic, control cotton plant.

Virus accumulation in transgenic and non-transgenic (susceptible), positive control plants was quantified by real-time PCR amplification, using primers specific to the DNA β-satellite CLCuMB, corresponding to a region not used for transgene construction. Betasatellite accumulation was negligible in the asymptomatic transgenic cotton plants, at 180–600 and 2500–15,590 molecules/µL for the T_0_ and T_1_ plants, compared to the symptomatic, non-transgenic, positive control plants, in which 4,015,249 CLCuMB molecules/µL were detected. Thus, the transgenic T_0_ and T_1_ generation plants, which exhibited no evidence of leaf curl symptoms post-virus inoculation, accumulated significantly less CLCuMB than symptomatic, non-transgenic control cotton plants included as the positive experimental controls e.g., susceptible to virus infection (Figure 3 and Figure 4). Also, for the T_0_ and T_1_ transgenic plant generations, the severity of disease symptoms, virus accumulation, and disease severity rating were positively correlated (Figure 5 and Figure 6).

### 3.5. Fluorescence In-Situ Hybridization Analysis

**Figure 7:**
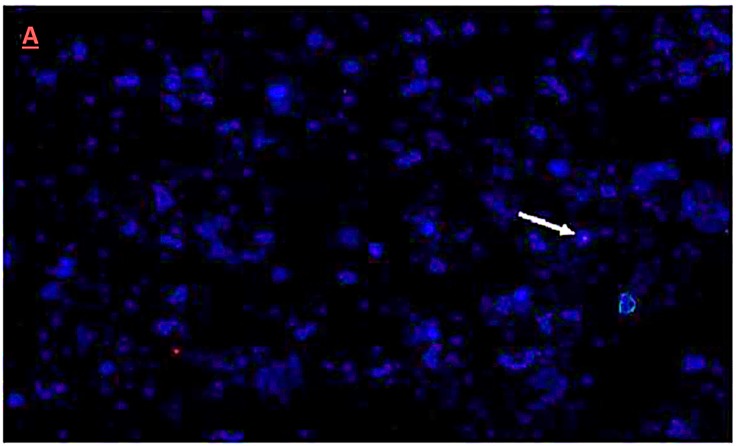

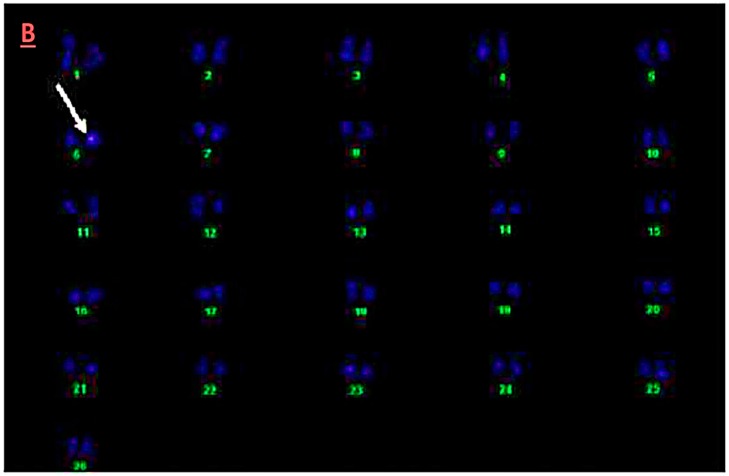
Fluorescence in situ hybridization (FISH) of the dsRNA hairpin construct in T_1_ generation plants. (**A**) Metastatic data for T_1_ transgenic cotton plants. The arrow indicates the location of transgene integration, as visualized by hybridization with a sequence-specific probe and fluorescent microscopy; (**B**) Karyotyping of a transgenic cotton plant Vβ6 in the T_1_ generation. The arrow indicates the location of the transgene on chromosome 6, visualized after chromosomes were re-ordered consecutively, using the karyotyping software Cytovision Genus version 3.93 Applied Imaging USA.

Integration and chromosome location in cotton plants of the dsRNA (hairpin) construct was confirmed by fluorescence insitu hybridization (FISH) and karyotyping, respectively. The presence of a single copy of the transgene was confirmed in six T_1_ transgenic cotton plants. The transgene for a selected plant of line Vβ6 that was asymptomatic and had the lowest relative virus accumulation among T_1_ generation plants was localized to chromosome 6 (Figure 7A,B).

### 3.6. Bioinformatic Analysis of the RNAi Construct

**Figure 8:**
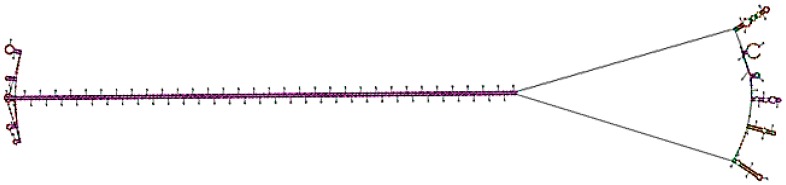
The RNA secondary structure of the Vβ construct, as predicted by the RNA structure tool.

Analysis of the dsRNA hairpin region of the construct using an RNA structure prediction tool [32], indicated the secondary structure of the molecule contained the loop and long, double-stranded RNA structure optimal for effective RNA-interference activity (Figure 8). The accuracy of prediction was determined to be 90%.

## 4. Discussion

In this study, the cotton variety “VH-289”, which is adapted to Pakistan growing conditions, was transformed with a dsRNA, anti-viral hairpin construct, referred to herein, as Vβ. The aim was to knockdown, or reduce the expression of the CLCuKoV-Bur *AV1* gene essential for viral replication initiation, and of the *βC1* gene and SCR region of CLCuMB, using the *Agrobacterium*-mediated embryo shoot apex cut method, as previously described [25,27]. The transgenic cotton plants harboring the Vβ construct showed substantially reduce betasatellite accumulation. The presence of the transgene construct was verified for cotton lines of the T_0_ and the T_1_ generations by PCR amplification of a ~300 bp fragment of the CLCuKoV*AC1* gene, which is essential for the initiation of begomoviral and betasatellite replication. FISH analysis and karyotyping of the Vβ6 plant, which was asymptomatic and had the lowest virus accumulation among the T_1_ generation plants, indicated the transgene was inserted as a single copy on chromosome 6. In contrast, *Agrobacterium*-mediated transformation can result in random integration of multiple transgene(s) of variable copy number [30].

Here, two successive generations of transgenic cotton plants were developed and evaluated for resistance to CLCuKoV-Bu and CLCuMB infection. Evidence for effective begomoviral-satellite knockdown was provided following challenge-inoculation of plants by viruliferous whiteflies, which resulted in the development of characteristic leaf curl symptoms in non-transgenic, positive control cotton plants, and absence of symptom development for all but one of the transgenic plant lines tested. Quantitative PCR analysis of the virus challenge-inoculated cotton plants indicated greatly reduced accumulation of the DNA β-satellite, CLCuMB, in the Vβ transgenic T_0_ and T_1_ generation plants, compared to non-transgenic cotton plants. For the non-transgenic susceptible and wild type virus-inoculated “control” plants, 2,367,884 molecules/μL and 4,015,249 molecules/μL of CLCuMB were detected, compared to extremely low levels for the T_0_ and T_1_ generation plants (Figure 3 and Figure 4), at 600 and 15,590 molecules/μL, respectively (Figure 3 and Figure 4). Finally, a positive correlation was observed between virus accumulation and disease severity (Figure 5 and Figure 6) in T_0_ and T_1_ generation plants. These results are comparable to a report by Asad et al. [10] in which siRNA sequences homologous to viral *AC1*, *AC2* and *AC3* coding regions were expressed in transgenic tobacco plants, resulting in ameliorated foliar symptoms, and another involving *G. hirsutum* “Coker 310” transgenic cotton plants expressing an antisense *βC1*, which showed reduced symptom severity [13]. Using the cotton leaf curl disease severity rating system of Akhtar and Khan 2002 [34], scores of 4 to 6 were assigned to non-transgenic cotton plants showing severe leaf curling, vein-thickening, enations, and stunting of plants, while transgenic plants were scored as 0 to 1, and developed mild or no symptoms. Thus, these results are also consistent with those of a previous study [35] that reported a positive relationship between begomovirus accumulation and symptom severity.

The durability of transgenic resistance mediated by RNAi for the simultaneous knockdown of begomoviral and betasatellite expression, remains to be further evaluated by subjecting plants to different CLCuKoV strains and inoculum levels, as well as closely-related leaf curl species that may share sequence homology in the viral and betasatellite regions targeted by this transgene construct. It may well protect against different species and strains, even in the absence of 100% shared homology, if the betasatellite sequences are sufficiently conserved. It is well known that betasatellites are promiscuous with respect to compatibility with multiple leaf curl strains and species, and so at the very least, this construct should act as a suppressor of host-mediated post-transcriptional silencing accordance with a well-known wild type function [19]. Nonetheless, the sequence-specific nature of RNAi would strongly suggest that these transgenic cotton plants can provide protection against multiple strains of CLCuKoV, including the widespread Burewala strain of CLCuKoV, currently the most prevalent begomoviral species infecting cotton in Pakistan.

## Figures and Tables

**Table 1:** Efficiency of cotton plant transformation with the Vβ construct.

Experiment Number	Seeds (*n*)	Embryos (*n*)	Embryos Transferred to MS Media (*n*)	Embryos Transferred to Glass Tube (*n*)	Plants Transferred to Greenhouse (*n*)	Plants in the Field (*n*)
1	20 g	10	10	0	0	0
2	20 g	30	28	28	5	1
3	20 g	45	38	20	6	1
4	20 g	70	70	12	1	0
5	40 g	100	95	15	8	3
6	20 g	35	30	0	0	0
7	40 g	132	132	10	7	4
8	20 g	10	10	10	2	1
9	20 g	33	33	14	5	3
**Total**	**220 g**	**465**	**465**	**109**	**34**	**13**

**Table 2:** Comparison of disease severity scores [1] for CLCuKoV-Bu-CLCuMB-inoculated T_0_ (generation 0) and T_1_ (generation 1) transformed and non-transformed cotton plants. The rating system is: 0 = no symptoms, 1–5 = incrementally, increasingly severe, and 6 = characteristic, severe leaf curl symptoms caused by *Cotton leaf curl* *Kokhranvirus*-Burewala/Cotton leaf curl Multan betasatellite (CLuKoV-Bu/CLCuMB) infection of susceptible cultivars.

**Control**	C-l	C-2	C-3	C-4	C-5	C-6	C-7	C-8	C-9
	6	6	4	4	4	5	5	6	5
**T_0_**	Vβ1	Vβ2	Vβ3	Vβ4	Vβ5	Vβ6	Vβ7	Vβ8	Vβ9
	0	0	0	0	0	0	0	0	0
**Control**	C1-1	C2-1	C4-1	C6-1					
	5	5	5	5					
**T_1_**	T_1_Vβ1	T_1_Vβ2	T_1_Vβ4	T_1_Vβ6					
	0	0	1	0					
